# Preliminary indications of the effect of a brief yoga intervention on markers of inflammation and DNA methylation in chronically stressed women

**DOI:** 10.1038/tp.2016.234

**Published:** 2016-11-29

**Authors:** K N Harkess, J Ryan, P H Delfabbro, S Cohen-Woods

**Affiliations:** 1 School of Psychology, The University of Adelaide, Adelaide, SA, Australia; 2 Cancer and Disease Epigenetics, Murdoch Childrens Research Institute, Parkville, VIC, Australia; 3 Department of Paediatrics, University of Melbourne, Parkville, VIC, Australia; 4 School of Psychology, Faculty of Social and Behavioural Sciences, Flinders University, Adelaide, SA, Australia

## Abstract

Yoga is associated with reduced stress and increased well-being, although the molecular basis for these benefits is not clear. Mounting evidence implicates the immune response, with current studies focused on protein immune markers (such as cytokines) in clinical populations. To explore the molecular impact, this pilot study uses a subsample (*n*=28) from a randomised waitlist control trial investigating the impact of an 8-week yoga intervention in a community population of women reporting psychological distress (*N*=116). We measured interleukin-6 (IL-6), tumour necrosis factor (TNF) and C-reactive protein (CRP) protein levels, and the DNA methylation of these genes and the global indicator, *LINE-1*. Correlations between these and psychological variables were explored, identifying moderate correlations with CRP protein levels, and methylation of *IL-6*, *CRP* and *LINE-1*. Many cytokine samples were below detection, however a Mann–Whitney *U* demonstrated a trend of moderate between-group effect for elevated IL-6 in the yoga group. Methylation analyses applied cross-sectional and non-controlled longitudinal analyses. Waist-to-height ratio and age were covaried. We demonstrated reduced methylation of the *TNF* region in the yoga group relative to the waitlist control group. No other genes demonstrated a significant difference. Longitudinal analysis further supported these results. This study is one of the first to explore yoga and immunological markers in a non-clinical population, and is the first study to explore DNA methylation. These findings indicate that further research into molecular impact of yoga on markers of immune function is warranted, with larger studies required.

## Introduction

Yoga is an increasingly popular technique combining physical activity, meditation and breathing practices (‘moving mindfulness'^1^), and is often practiced as a treatment/adjunct treatment for psychiatric conditions.^2^ A growing body of psychological literature demonstrates that practicing yoga improves subjective well-being and positive feelings, and reduces reported levels of stress, distress and negative feelings, including clinical symptoms of depression and anxiety.^3, 4, 5^

Inflammation has been demonstrated to be associated with depression and exposure to stressors, specifically including the action of the inflammatory cytokines interleukin-6 (IL-6) and tumour necrosis factor (TNF^6, 7, 8, 9^) and the acute-phase protein C-reactive protein (CRP^10, 11, 12^). Further, these have been postulated to be impacted by both exercise and psychological therapies. Anti-inflammatory factors are modified by participation in moderate exercise,^13^ and with participation in a mindfulness-based stress reduction intervention.^14^ Biochemical evidence indicates practices such as yoga reduce inflammatory responses associated with stressful situations.^15, 16^ Our current understanding of the molecular mechanisms involved in the modulatory effect of yoga remains limited however.

Inflammation changes reported in the literature may, in part, be determined by epigenetic processes that impact gene expression, and ultimately protein expression. The epigenome regulates gene expression, and can be altered by environmental factors such as stress.^17^ Epigenetic changes are increasingly recognised as relevant biomarkers for mental illness, with DNA methylation the most widely studied.^18, 19, 20, 21^ The changes in DNA methylation have been associated with poor physical health, and high levels of inflammation.^22, 23, 24, 25^ As epigenetic changes are potentially reversible, they may be used for the evaluation of responses to clinical therapies.^26^

Emerging studies of mind-body therapies (MBTs), including yoga-based interventions, are increasingly exploring mechanisms;^27, 28, 29, 30^ however, most studies focus on gene-expression changes.^31^ Thus while a change in gene expression, and therefore a biological effect may be reported, the mechanism of this effect remains unknown. Only two epigenetic studies currently exist in the MBT literature and indicate that interventions conceptually similar to yoga may be correlated with epigenetic change. Specifically, an 8 h meditation session has been reported to rapidly alter global modification of histones, and reduce expression of histone deacetylase and pro-inflammatory genes.^32^ DNA methylation changes in six age-related CpG sites have also been reported in a cross-sectional study of Australian female long-term tai chi practitioners.^33^ However, no studies have investigated the relationship between a psychophysiological intervention, such as yoga, on indicators of genome-wide DNA methylation (which can be explored broadly utilising a repetitive element sequence as a surrogate, such as LINE-1; ref. 34), and DNA methylation patterns of immune candidate genes such as *TNF*, *IL6* and *CRP*, candidates implicated in psychological distress and to be altered by mindfulness-based stress reduction and yoga practice. DNA methylation in these genes have been investigated in the context of inflammatory conditions (rheumatoid arthritis) and engagement in physical activity, age, pollution exposure and weight-related factors.^34, 35, 36, 37, 38, 39, 40, 41^ Although findings have been mixed, they have demonstrated that DNA methylation changes are observed in relation to physical factors and across relatively short time periods.

The objective of this pilot study is twofold: (1) to examine the epidemiological effect of a yoga intervention on markers of inflammation (IL6, TNF and CRP); and (2) to examine, for we believe the first time, whether participation in a yoga intervention (an MBT) is associated with altered levels of estimated global DNA methylation (represented by methylation of the interspersed repeat *LINE-1*) or changes to methylation patterns of the *IL6*, *TNF* and *CRP* genes. Specifically, we have conducted a longitudinal analysis on protein markers of inflammation, comparing distressed middle-aged women who have engaged in a 2-month yoga intervention with a waitlist control group. Second, we have conducted a cross-sectional analysis of between-group DNA methylation profiles comparing post-yoga intervention group with the waitlist group. Finally, we have conducted a longitudinal analysis of the waitlist group's DNA methylation profiles to corroborate the cross-sectional analysis.

## Materials and methods

### Participants and procedure

This study represents a subsample (*n*=28) of a larger clinical trial (*N*=116), which explores the psychophysiological effects of a yoga intervention in women reporting psychological distress (as measured by a score of 16+ on Kessler Psychological Distress Scale [K10], ref. 42) and utilises a stratified, randomised waitlist control trial design. Psychophysiological results are reported elsewhere.^43^ The parent study explores the psychophysiological effects of participation in an average of a 1 h yoga class per week for a period of 8 weeks. The study utilised a stratified, randomised waitlist control trial design (described in detail in ref. 44). Within the parent study, a subsample of participants were randomly allocated to provide serum samples for the analysis of cytokines (IL-6 and TNF) and high-sensitivity CRP (*n*=35; *n*=7 lost to follow-up). Women were eligible for this pilot study if they were: healthy, free from acute infection for 2 weeks before biochemical assessment, and if they had refrained from drinking alcohol in the 48 h before biochemical assessment. Additional exclusion criteria were serious physiological illnesses that would interfere with the interpretation of biochemical data (for example, anaemia, diabetes, cardiovascular diseases, blood cancers, inflammatory bowel diseases, autoimmune diseases, asthma being treated with steroids, immunodeficiency); having a body mass index >30; meeting the criteria for substance abuse or dependence; undergoing menopause; having a serious psychological illness; or, having engaged in a regular yoga practice within the previous year. Biological samples were only available for this subsample.

Selection for epigenetic analysis in this subgroup is based on participants (1) already consented to provide blood; (2) meeting the inclusion/exclusion criteria described; and (3) giving informed consent to their blood sample being used for genetic analysis before the post-treatment evaluation. The participants who fulfilled the first two criteria were identified and randomly allocated into this portion of the study using Research Randomizer.^45^ The mean age of participants in this subsample (*M*=41.21, s.d.=4.14) is younger than the parent study (*M*=48.14, s.d.=8.22), but participants are not appreciably different in terms of other demographic or clinical variables. This trial has been approved by the Human Research Ethics Committee of the University of Adelaide; all the participants have given informed consent. This trial is registered at the Australian New Zealand Clinical Trial Registry [ANZCTR]: ACTRN12616000612415.

### Yoga intervention

The yoga intervention comprised 8 weeks of twice-weekly, hour-long yoga classes (the total number of classes offered was 16). Per-protocol completion was considered attendance at eight classes, as weekly practice reflects the average community practitioners' engagement.^2, 46^ For further details, see Harkess *et al.*^44^

### Study design

**Figure 1:**
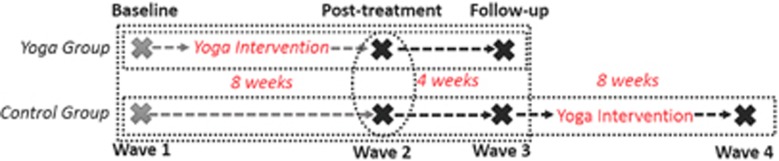
A visual depiction of the parent study and the current sub-study to explicate the analyses conducted. Grey ‘X' markings depict where only serum samples were available for the analysis (inflammatory markers), and black ‘X' markings indicate that both serum and whole bloods were available for the analysis (inflammatory markers and DNA methylation). The perforated rectangles indicate the longitudinal analyses conducted (where possible), and the perforated oval indicates the conduct of cross-sectional analysis.

The study analyses involve two parts. The first utilises a randomised trial design to compare protein markers of inflammation of the participants who completed the yoga intervention to those of the control group (IL-6, TNF and CRP). The second utilises a cross-sectional trial design to compare DNA methylation patterns of participants who completed the yoga intervention to those of the control group at the post-treatment assessment. This is due to consent and ethics for genetic analysis being granted after initiation of the study, but before post-treatment data and sample collection. DNA methylation patterns are also explored longitudinally in a non-controlled trial design, with the waitlist control group examined from post-treatment and follow-up time points, until after the completion of the second round of yoga classes utilising our standardised protocol. To avoid confusion, we will refer to these as ‘waves' (see Figure 1).

### Sample collection

Assessment included completing online surveys including demographic and psychological variables (detailed below), which participants completed before an in-person assessment. The in-person assessment involved physiological tests (that is, waist and height measurements) and collection of blood samples through routine venepuncture at baseline (wave 1), post-test (wave 2), 1-month follow-up (wave 3) and waitlist control intervention post test (wave 4). The participants were requested to abstain from stimulants, such as coffee, on the day of testing. At wave 1, the phlebotomist drew 21 ml intravenous blood sample from each participant. Each sample provided 3 ml for a complete blood picture analysis (to screen samples for abnormalities), 9 ml for cytokine analysis and 9 ml for hsCRP analysis. At waves 2, 3 and 4, the phlebotomist drew a total of 30 ml, with 9 ml extra to allow for genetic analysis. VACUETTE Plastic K3EDTA tubes (purple top) were used for complete blood picture and genetic analysis of samples, and VACUETTE Z Serum Sep Clot Activator (white top) were used for cytokines and hsCRP. The complete blood picture was analysed on the day of testing. To avoid problems with assay drift and interassay variability, samples for hsCRP and cytokines were centrifuged as per manufacturer's protocol, and the serum was frozen at −80 °C until the study was completed (post wave 4). For DNA analysis, whole blood samples were aliquoted into seven to eight eppendorfs, each containing 1 ml volume of whole blood and stored at −80 °C for DNA extraction and analysis as required. The remaining 1 ml was stored in RNAlater (Life Biosciences, Thermo Fisher, Vilnius, Lithuania) and is stored at −20 °C for future gene-expression analysis.

#### Mental health variables

As a set of secondary analyses, we also explored associations between biochemical outcomes (protein and DNA methylation inflammatory candidate markers) and psychological variables that have already demonstrated between-group effects in this population (Harkess *et al.*, in press). Specifically, the study explores outcome scores at post-test on the (a) Kessler Psychological Distress Scale (K10), which gives a global measure of psychological distress based on questions about anxiety and depression symptoms;^42^ and the (b) Perceived Stress Scale, which measures the degree to which situations in one's life are appraised as stressful;^47^ and Positive Affect of the Positive and Negative Affect Schedule, which is a mood scale that measures people's positive affect.^48^

#### Protein analysis

Biological analyses were conducted blind of treatment/control groupings, with individuals across groups included in any batch to prevent group-specific batch effects. The study determined hsCRP serum concentration using the Beckman Coulter AU2700 analyser (Olympus, Hamburg, Germany; Beckman Coulter, Krefeld, Germany) and the Beckman Coulter CRP Latex method (immune-turbidimetric test), following the manufacturer's recommended protocol. A highly sensitive application that has a dynamic range of 0.08 to 80 mg l^−1^ was used. The samples from all the four waves were run by one individual in a batch of 20–30 over 2 days. The calibration was performed as required and quality control samples were run in accordance with SA Pathology protocols (internal quality controls were reported to be between 7–9% at the time of analysis).

Cytokine (IL-6 and TNF) serum concentrations were measured by cytokine capturing beads, using the BD cytometric Bead Array Human Enhanced Sensitivity Master Buffer kit and following the manufacturer's recommended protocols. Sensitivity of this kit is reported between the range of 0.27 to 200 pg. The samples were analysed by flow cytometry on the BD Canto1 flow cytometer. Quality control was performed daily, using Cytometer Setup and Tracking beads and an assay utilising the reported kit to determine whether proper cytokine readings were taken. A number of samples demonstrated levels below the 0.274 pg threshold for detection (IL-6: wave 1=11; wave 2=10; wave 3=10; wave 4=6; and TNF: wave 1=16; wave 2=16; wave 3=15; wave 4=7).

#### Methylation analysis

Biological analyses were conducted blind of treatment/control groupings, with individuals across groups included in any batch to prevent group-specific batch effects. Methylation assays were designed with Epidesigner software (www.epidesigner.com) and covered key regions found to be differentially methylated in previous studies investigating other exposures or disease outcomes: *TNF*;^37, 40, 41, 49, 50^ *IL6*;^35, 36, 38, 39, 40^ previously reported LINE-1 primers;^51^ and, the *CRP* assay was designed to target the CpG sites in the promoter region. (See Supplementary Table 6 for the assay designs) Cleavage patterns were determined using the Bioconductor MassArray package in R (www.bioconductor.org). DNA was extracted using the QIAamp DNA Mini Kit (QIAGEN, Hilden, Germany), and bisulphite converted using the MethylEasy Xceed Kit (Genetic Signatures, Darlinghurst, NSW, Australia). The samples were PCR amplified and assayed in triplicate. DNA methylation was quantified using the SEQUENOM MassARRAY (San Diego, CA, USA) and methylation ratios calculated using EpiTyper software (v.1.2; SEQUENOM). Further PCR protocol details and conditions are included in Supplementary Materials (Supplementary Tables 7).

The mean methylation from three technical replicates for each sample was determined; outlying values (deviation of ±10% methylation from the median) were discarded. Any individual with only one methylation datapoint following outlier identification was excluded from cross-sectional analyses. In longitudinal analysis, discarding these individuals limited the sample size with multiple datapoints (that is, sample was <6), so we retained single methylation datapoint individuals for the purpose of this pilot study (*n*=10), with sensitivity analyses excluding these individuals in the Supplementary Data (Supplementary Tables 1).

#### Statistical analysis

Statistical analyses were conducted using SPSS for Windows, version 21, software (SPSS, Chicago, IL, USA). The non-normal IL-6 and TNF distributions were dealt with by utilising non-parametric statistical tests.

#### IL-6 and TNF protein marker analysis

With no non-parametric equivalent to a two-way analysis of vairance (ANOVA), we used two Friedman Tests for longitudinal analyses to investigate change over time within each group (yoga and waitlist control groups, separately), which allowed use of all the available data within the study: to investigate change over time in waves 1, 2 and 3 in the yoga group (analyses 1); to investigate change over time in waves 1, 2, 3 and 4 in the waitlist control group (yoga was engaged in with the waitlist control group between waves 3 and 4; analyses 2). To compare the between-group differences post intervention at wave 2 on IL-6 and TNF protein levels, a cross-sectional analysis was applied: Mann–Whitney *U*-tests were used (analyses 3).

#### hsCRP analysis

(Analyses 4) A mixed between–within subjects ANOVA was conducted to assess the impact of the yoga intervention on hsCRP levels. This included data from waves 1, 2 and 3 for both yoga and waitlist control groups. (Analyses 5) A one-way repeated-measures ANOVA was used to investigate whether change over time was observed for hsCRP in the waitlist control group following yoga exposure; this included three waves before yoga (1, 2 and 3) with the final wave post yoga (wave 4).

#### DNA methylation

For each immune candidate (*IL6*, *TNF*, *CRP*), a mean percentage of methylation was calculated across all the CpG sites in each region assayed. Two sets of analyses were conducted: (analyses 6) an analysis of covariance model was used to evaluate cross-sectional outcome measures for DNA methylation data, with yoga as the predictor (no covariates). A second analysis of covariance was run to control for potential confounders (age and waist-to-height ratio at wave 2). We conducted two analyses due to the small sample size (*N*=28) and the exploratory nature of this study. Utilising the two analyses allows examination of the impact of additional covariates on the F-value, which is sensitive to degrees of freedom. (Analyses 7) To evaluate change across time following yoga intervention, we were restricted to utilising the waitlist control group only for longitudinal analysis as we did not have DNA methylation data for wave 1; a *t*-test was conducted, with the mean of the two pre-intervention results (wave 2 and wave 3) compared with post-intervention methylation (wave 4).

#### Secondary exploratory analyses of measures of mental health

(Analyses 8) We were restricted to correlational analyses owing to insufficient numbers to enable regression-based analyses.^52^ The protein biomarkers, IL-6 and TNF, exhibit non-normal distributions, thus non-parametric correlational analyses were applied. A Spearman rank-order correlation was performed to explore post-test associations of inflammatory protein markers, DNA methylation and mental health outcome variables.

#### Effect size and significance

As recommended by Perneger,^53^ we discuss the results in regard to both statistical significance and effect size (where possible), specifically Spearman's *r* (small=0.10, medium=0.30, large=0.50), partial eta squared (*η*_ρ_^2^; small=0.01, medium=0.06, large=0.138) and Cohen's *d* (small=0.02, medium=0.50 and large=0.80).^54^ Each hypothesis has been considered individually.^53^

### Code availability

Computer code used to analyse the data can be made available by email upon request.

## Results

### Characteristics of the participants

As displayed in Table 1, the main characteristic of the participants did not differ significantly between the yoga and control groups, including in energy expenditure (METs), indicating equal engagement in physical activity. All the participants who participated in the blood sampling were Caucasian; therefore, we did not control for ethnicity.

### Analysis of inflammatory markers

#### Analyses 1

The Friedman Test indicated there was no evidence of a longitudinal difference in IL-6 or TNF across the three time points (*X*^2^ (2, *n*=11)=2.34, *P*=0.310; *X*^2^ (2, *n*=11)=0.50, *P*=0.779). Analyses 2: the Friedman Test indicated there was no evidence of a difference in IL-6 or TNF across the four time points (*X*^2^ (3, *n*=9)=0.57, *P*=0.904; *X*^2^ (3, *n*=9)=2.10, *P*=0.551). Analyses 3: a Mann–Whitney *U*-test revealed a nonsignificant, but moderate effect size suggesting that at post-test IL-6 levels were higher in the intervention group (*M*_d_=1.33, *n*=11) than in the control group (*M*_d_=0.00 *n*=15; *U*=49.0, *z*=−1.79, *P*=0.073, *r*=0.35). There was no evidence for differences in TNF levels (non-detectable intervention: *M*_d_=0.00, *n*=11; control: *M*_d_=0.00, *n*=15; *U*=78.0, *z*=−0.27, *P*=0.790, *r*=0.05).

#### Analysis 4

A mixed between–within subjects ANOVA demonstrated nonsignificant effect, but good effect size for differences in CRP over time, Wilks' Lambda=0.75, F(2,19)=3.17, *P*=0.065, *η*_ρ_^2^=0.25; though there was no evidence of a group by time interaction, Wilks' Lambda=0.91, F(2,19)=0.91, *P*=0.421, *η*_ρ_^2^=0.09. The means and standard deviations are presented in Table 2. Analysis 5: a one-way repeated-measures ANOVA (analysis 5) indicated there was no effect for time, Wilks' Lambda=0.54, F(3,5)=1.42, *P*=0.342, *η*_ρ_^2^=0.46. The means and standard deviations are presented in Table 3.

### Analysis of DNA methylation

Please see Table 4 for depiction of the cross-sectional analysis (wave 2; analysis 6), described below.

#### Regions of methylation and yoga

No between-group differences in mean methylation across *CRP* or the *IL6* regions were observed in either analysis of covariance model (no covariates, or with age and WtHR as covariates). A significant main effect of group was found for the mean methylation of *TNF*, which explained 19% of the variance. Women in the yoga group demonstrated a 4.5% lower level of methylation relative to the waitlist control group (see Table 4). The main effect of the group on *TNF* remained (yoga group with lower methylation) when covariates age and waist-to-height ratio were included in the analysis.

#### Individual CpG units and yoga

No significant differences in methylation at individual CpG units were demonstrated for *CRP* and *IL6*_2;_, differences in mean methylation for *IL6*_1_ CpG site 1 were observed between groups (2.3% higher methylation in the yoga group), but this association was reduced with the inclusion of age and WtHR in the model. There appeared to be some differences between groups at individual *TNF* sites, but this varied depending on the inclusion of covariates. Only one covariate, WtHR, demonstrated a close to significant association with the *TNF* CpG site 4/5/6 (*P*=0.050; 20% of variance explained).

#### Global DNA marker LINE-1 methylation

No evidence for differences in methylation at individual *LINE-1* CpG units, nor the overall mean, was demonstrated. Covariates were also not associated with differences in *LINE-1* methylation.

**Figure 2:**
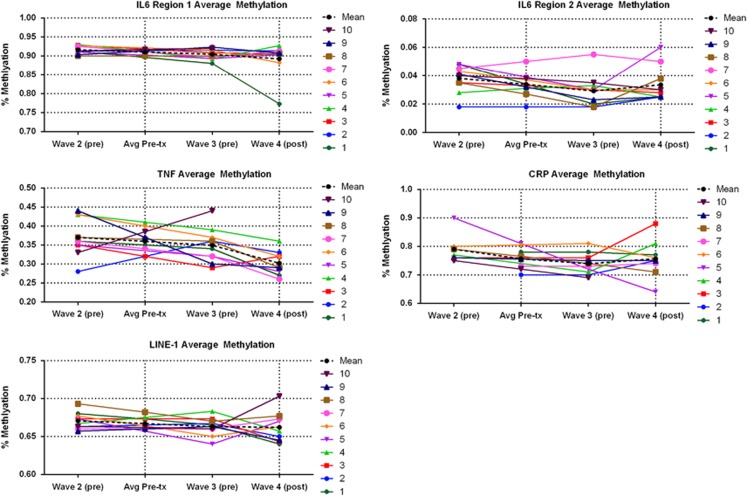
Longitudinal IL-6 (regions 1 and 2), TNF, CRP and global DNA marker LINE-1 methylation patterns of the control group. Each participant is shown individually (numbered 1–10), with the mean shown as a black perforated line. (Avg Pre-tx indicates average methylation pre-yoga intervention at waves 2 and 3; pre indicates pre-yoga intervention; post indicates post-yoga intervention). CRP, C-reactive protein; IL-6, interleukin-6; TNF, tumour necrosis factor.

Please see Figure 2 for the depiction of longitudinal analysis of the waitlist control group (analysis 7). The sample sizes for the longitudinal analyses are small (ranging from 10 to 11). As some longitudinal techniques used to compare the groups are unreliable in the small sample sizes, we conducted paired sample *t*-tests to explore pre- to post-intervention (average of wave 2 and 3 to wave 4) effects to ascertain whether findings corroborated cross-sectional analyses already presented. The results of all the analyses and descriptive statistics are presented in the Supplementary Material (reference Supplementary Tables 1–5), and we present psychological outcomes that demonstrated medium or large effects of change following yoga.^54^

#### Regions of methylation

The yoga intervention was associated with a reduction in *TNF* methylation (Cohen's *d*=1.68) and decreased *IL6*_*1*_ methylation (Cohen's *d*=0.53), although this did not reach significance.

#### Individual CpG units

Significant associations indicating decreased methylation at the post-yoga time point was demonstrated for *TNF* CpG site 1 (Cohen's *d*=1.11) and 4/5/6 (Cohen's *d*=1.00).

#### Global DNA marker LINE-1 methylation

No evidence of a difference for time was seen at individual *LINE-1* CpG sites or for the mean.

### Exploratory analyses of measures of mental health

#### Analyses 8

As shown in Table 5, there is a strong correlation between perceived stress and psychological distress. Moderate correlations are demonstrated between subjective well-being and perceived stress and psychological distress. A moderate and significant correlation between global DNA marker *LINE-1* methylation and perceived stress is reported. A number of other moderate size correlations are observed, however, significance was not achieved, possibly due to limited power.

## Discussion

This prospective pilot trial explored the relationship between yoga, psychophysiological health indicators, and inflammatory protein and methylation markers in a stressed female community population. This study was unique in exploring DNA methylation, and correlations between methylation and inflammatory markers with potential to indicate a functional relationship. The DNA methylation component was, however, included retrospectively, meaning longitudinal analysis was only possible with the waitlist participants pre- and post-yoga intervention. Overall, the study found that an 8-week yoga intervention, requiring at least weekly practice, is associated with some changes in immune protein and DNA methylation biomarkers. The yoga group demonstrated lower DNA methylation of the *TNF* region as a whole, and at specific sites, in cross-sectional analysis relative to the control group. This was further supported by decreased methylation seen post yoga in the longitudinal analysis of the waitlist control group that later participated in the yoga intervention. Meaningful effects sizes in both protein and methylation analysis were demonstrated, as were associations between psychological variables and biochemical measures; however, these were not found to be significant. Lack of significance may be attributed to limited statistical power of the study. Nonetheless, these results indicate that participation in an 8-week yoga intervention may have differential impacts on the methylation responses of the immune candidate genes investigated, and that further investigation in better-powered samples is important.

Of note, we did not find evidence of associations between yoga and serum measures of inflammation. Similarly, a large-scale trial did not demonstrate an association between anxiety and biomarkers of inflammation in females,^55^ which contrasts the associations reported in depression.^56, 57^ To this end, it should be considered that this is a non-clinical community population in which biomarkers of inflammation were generally low, reflected by the ‘bottoming out' of inflammatory cytokines. Nonetheless, the moderate effect indicating higher IL-6 levels in the yoga group is interesting insofar as it has a well-known role in the pro-inflammatory processes, but is increasingly recognised in healing and regeneration activities.^58^ For instance, Eyre *et al.* reports that increased IL-6 has a role in the neuroprotective effect of exercise on mood.^59^ A large effect for time on overall levels of hsCRP was demonstrated, though we did not find evidence of meaningful between-group difference. The general decrease at post-test may be reflecting a sample bias we have discussed elsewhere.^43^ Namely at baseline, women were reporting chronic stress and moderate-to-high levels of distress (potentially indicated by high levels of acute-phase proteins), however, they self-selected for this study, which indicates motivation to change. This was supported by the overall decrease in stress and distress at post-test,^43^ and could account for a change in hsCRP over time independent of participation in yoga. In addition, at post-test, perceived stress was found to be positively associated with global DNA marker *LINE-1* methylation. Although this is not consistent with some literature, indicating elevated methylation correlates with positive health outcomes,^60, 61^ it is consistent with literature demonstrating hypermethylation in stressed populations.^62^

This study reports a robust association between engagement with an 8-week yoga intervention and reduction in mean methylation of *TNF* (5.5%); however, there is no evidence for sizeable correlations between the *TNF* methylation and serum, or psychological measures making it difficult to infer causal relationships. We do, however, report a moderate association with WtHR, parsimonious with previous reports of methylation of *TNF* being associated with leanness/weight-loss previously.^40, 41^ This could potentially account for our reported hypomethylation of *TNF* in our yoga group and following yoga intervention. However, including WtHR as a covariate did not alter the reported association; in fact, it strengthened it suggesting that the reduction observed in *TNF* methylation is not simply attributable to change in body composition in our sample following yoga. Yoga may be associated with a positive alteration on the inflammatory system that is not detectable immediately in serum analysis, and not directly responsible for reported positive psychological effects of yoga.

This sample was relatively homogeneous in variables that have been reported as risk factors for differential global DNA marker of methylation (as measured by LINE-1; for example, see refs 60,61,63,64,65,66,67,68,69). Namely, it comprised Caucasian females, aged between 35 and 50 years, with body mass indexes <30, with no reported substance abuse problems, and comparable between-group physical activity levels (no between-group differences in METS discussed in greater detail elsewhere; for example, ref. 43). The yoga and control group were overarchingly well matched for WtHR and age. Inclusion of WtHR, which was associated with *TNF* methylation at CpG 4/5/6, as a covariate improved detection of differences in *TNF* methylation following our yoga intervention. Thus, although the lack of statistical correlation and between-group effects could be attributable to the limited statistical power in our sample, the medium to large effect sizes reported are plausibly attributable to the yoga intervention, and not to previously implicated lifestyle factors due to a lack of variability of these factors in our groups (that is, ethnicity, WtHR, age). This study presents strengths for investigation of biological biomarkers, in the selection of an homogenous population, longitudinal sampling and the first study to investigate *DNA* methylation in context of a yoga intervention. However, there are limitations.

### Limitations

There are a number of limitations to this study, which should be considered. The first is that it was a pilot study with only limited statistical power. As a result, this limited statistical analyses that could be undertaken (for example, we did not meet the sample size assumption required to conduct a regression, nor exploration of mediation/moderation), in addition to having low power when measuring the group differences (for example, ANOVA cell sizes of 30 are required for 80% power^70^). Second, there was no baseline DNA methylation measure, which means we cannot draw causal conclusions about between-group differences. DNA methylation investigated was from DNA extracted from peripheral whole blood, which is in keeping with our simultaneous exploration of serum markers of inflammation. However, we cannot draw inferences about the effects on specific tissues, including the brain. We only explored a limited number of immune candidate genes (two regions of *IL6* and one region of *TNF* and *CRP* each), and, as demonstrated by *IL6*, different regions may indicate different trends. The lack of association between DNA methylation markers and serum markers of inflammation makes it difficult to interpret functional impact, although this could be due to statistical power and the possibility that methylation impacts less immediately in serum protein expression levels. It is notable that a number of cytokine samples were below the detection limit in protein analyses, which is likely due to the non-clinical nature of this sample as well as a technical limitation of the sensitivity of currently available assays. We did not use an active control group, and while between-group METs were equivalent, we cannot rule out the attentional effects of engagement with the yoga teacher and the class environment. To address some of these concerns, we focused on the presentation of effect sizes where available and also analysed the data from two different perspectives to examine the reliability of the findings (cross-sectional and longitudinal).

As levels of stress are reported to be increasing in community populations,^52^ an increased prevalence of stress-related disease is likely to follow.^6, 71, 72, 73^ Therefore, future prospective studies should continue to explore the relationship of stress and biomarkers of inflammation in community populations. We recommend replicating our study in a much larger sample and including analysis of DNA methylation profiles at baseline. A variety of active controls would also be beneficial to assist in disentangling the potentially different effects of different styles of yoga, exercise and meditation. In addition, we recommend exploring other candidate genes that may demonstrate involvement in the inflammatory response that has been associated with maladaptive psychological states and/or epigenomic methods that enable network analyses. Finally, we would recommend an experimental design that could differentiate more clearly between regressions to the mean (that is, entering the study when distress levels are maximal and a natural decrease with time as opposed to intervention) and an experimental effect would be one that took a number of pre-intervention measures.

## Conclusions

Alongside the increased levels of stress and prevalence of stress-related disease reported, there has been increased engagement in MBTs, of which yoga is the most utilised.^74^ Although gene-expression studies in the MBT literature suggests a relationship with the immune system,^75^ further research into the underlying mechanisms, including possible epigenetic mechanisms, has been called for.^31^ To the best of our knowledge, this is the first study to investigate the role of yoga on epigenetic change, and the first MBT to investigate DNA methylation in immune candidates' methylation (*IL6, TNF* and *CRP*). Although this pilot study is small and exploratory, it nevertheless indicates that in a non-clinical chronically stressed community population, practicing a minimum of a once-weekly, hour-long yoga class, is associated with differential methylation patterns despite the waitlist control group reporting similar energy expenditure to the yoga group. This suggests that these changes may not be related to energy expenditure, but some aspect of the yoga engagement. However, it is notable that the control group shows larger variance in energy expenditure relative to the intervention group, and more definitive conclusions cannot be made without an active control group in future studies. Specifically, we report that engaging in a yoga intervention may affect the female participants' serum levels of IL-6 and their epigenetic profile of immune candidates, specifically TNF. These findings warrant further large-scale research and contribute to the growing literature seeking to explore underlying epigenetic mechanisms and the relationship between MBT, the immune system.^31^ In addition, they contribute to the growing body of literature seeking to explore biomarkers of inflammation in the clinical and non-clinical conditions of distress.^76, 77, 78^

## Figures and Tables

**Table 1:** Baseline participant characteristics

	*Overall (*N*=26)*		*No.*	*%*	*Control (*N*=15)*		*No.*	*%*	*Yoga (*N*=11)*		*No.*	*%*
	*Mean*	*s.d.*			*Mean*	*s.d.*			*Mean*	*s.d.*		
Age (years)	41.12	4.28			40.80	4.36			41.55	4.34		
Waist-to-height	0.50	0.07			0.48	0.08			0.52	0.07		
BMI	24.78	4.96			24.20	5.38			25.57	4.46		
METS	3162.00	7987.60			4080.40	10 515.93			1909.64	1302.00		
K10	23.69	5.22			24.20	5.45			23.00	5.06		
WBCC^a^	6.29	1.59			6.28	1.04			6.32	2.19		
hsCRP^a^	1.43	1.23			1.73	1.45			1.02	0.73		
												
*Marital status*												
Single			4	15.4			2	13.3			2	18.2
Common-law/married			17	69.2			10	66.7			8	72.7
Separated/divorced			3	11.5			2	13.3			1	9.1
Declined to answer			1	3.8			1	6.7				
Parous			16	61.5			7	46.7			8	72.7
												
*Education level*												
High school or less			4	15.3			1	6.7			3	27.3
Vocational school			4	15.4			2	13.3			2	18.2
University graduate			14	53.8			9	60			5	45.5
Postgraduate			4	15.4			3	20			1	9.1

Abbreviations: BMI, body mass index; hsCRP, high-sensitivity C-reactive protein; K10, psychological distress; METS, metabolic equivalent; WBCC, white blood cell count.

a Levels are reported in untransformed units.

**Table 2:** Descriptive statistics for hsCRP for varying time points

	*Yoga intervention group*		*Waitlist control group*		*Total/combined*	
	N	*Mean (s.d.)*	N	*Mean (s.d.)*	N	*Mean (s.d.)*
Baseline (wave 1)	10	1.00 (0.76)	12	1.49 (1.38)	22	1.27 (1.14)
Post-test (wave 2)	10	1.05 (0.81)	12	0.99 (0.49)	22	1.02 (0.64)
Follow-up (wave 3)	10	1.79 (1.59)	12	1.50 (1.24)	22	1.63 (1.38)

Abbreviation: hsCRP, high-sensitivity C-reactive protein.

**Table 3:** Descriptive statistics for hsCRP for pre-intervention time points and post-test

*Time period*	N	*Mean*	*s.d.*
Pre-test (wave 1)	8	1.56	1.67
Pre-test (wave 2)	8	0.84	0.29
Pre-test (wave 3)	8	1.45	1.49
Post-test (wave 4)	8	1.00	0.96

Abbreviation: hsCRP, high-sensitivity C-reactive protein.

**Table 4:** Results of DNA methylation cross-sectional ANCOVA analyses

*Promoter region*	*Control*		*Yoga*		*Main effect*	*Effect with covariates* *(age and WtHR)*	
	*Mean (s.d.)*	n	*Mean (s.d.)*	n	*Yoga vs control*	*Yoga vs control*	*Covariates*
*IL6*_*1*_							
CpG 1	0.898 (0.024)	13	0.921 (0.021)	9	F(1,20)=5.29, *P*=0.032*, *η*_ρ_^2^=0.21	F(3,18)=4.30, *P*=0.053^†^, *η*_ρ_^2^=0.19	Age *η*_ρ_^2^=0.11, WtHR *η*_ρ_^2^=0.02
CpG 2/3	0.931 (0.013)	10	0.924 (0.019)	8	F(1,16)=0.768, *P*=0.394, *η*_ρ_^2^=0.05	F(3,14)=1.12, *P*=0.307, *η*_ρ_^2^=0.07	Age *η*_ρ_^2^=0.07, WtHR *η*_ρ_^2^=0.04
CpG 4/5/6	0.928 (0.016)	14	0.933 (0.009)	9	F(1,21)=0.59, *P*=0.452, *η*_ρ_^2^=0.03	F(3,19)=0.27, *P*=0.609, *η*_ρ_^2^=0.01	Age *η*_ρ_^2^=0.02, WtHR *η*_ρ_^2^=0.11
Mean	0.853 (0.246)	14	0.926 (0.011)	9	F(1,21)=0.78, *P*=0.387, *η*_ρ_^2^=0.04	F(3,19)=0.72, *P*=0.406, *η*_ρ_^2^=0.04	Age *η*_ρ_^2^=0.10, WtHR *η*_ρ_^2^=0.00
							
*IL6*_*2*_							
CpG 1	0.035 (0.009)	15	0.037 (0.010)	11	F(1,24)=0.24, *P*=0.626, *η*_ρ_^2^=0.01	F(3,22)=0.14, *P*=0.717, *η*_ρ_^2^=0.00	Age *η*_ρ_^2^=0.02, WtHR *η*_ρ_^2^=0.04
CpG 2	0.006 (0.009)	15	0.003 (0.005)	11	F(1,24)=0.91, *P*=0.349, *η*_ρ_^2^=0.04	F(3,22)=1.22, *P*=0.281, *η*_ρ_^2^=0.05	Age *η*_ρ_^2^=0.05, WtHR *η*_ρ_^2^=0.04
CpG 4/5/6	0.033(0.011)	15	0.034 (0.009)	11	F(1,24)=0.04, *P*=0.852, *η*_ρ_^2^=0.00	F(3,22)=0.01, *P*=0.931, *η*_ρ_^2^=0.00	Age *η*_ρ_^2^=0.04, WtHR *η*_ρ_^2^=0.00
CpG 7/8	0.078(0.020)	15	0.084 (0.015)	11	F(1,24)=0.56, *P*=0.463, *η*_ρ_^2^=0.02	F(3,22)=0.44, *P*=0.515, *η*_ρ_^2^=0.02	Age *η*_ρ_^2^=0.00, WtHR *η*_ρ_^2^=0.01
Mean	0.038 (0.008)	15	0.040 (0.007)	11	F(1,24)=0.13, *P*=0.720, *η*_ρ_^2^=0.01	F(1,22)=0.05, *P*=0.824, *η*_ρ_^2^=0.00	Age *η*_ρ_^2^=0.04, WtHR *η*_ρ_^2^=0.03
							
*TNF*							
CpG 1	0.829 (0.079)	13	0.748 (0.108)	6	F(1,17)=3.45, *P*=0.081^†^, *η*_ρ_^2^=0.17	F(3,15)=2.32, *P*=0.148, *η*_ρ_^2^=0.13	Age *η*_ρ_^2^=0.04, WtHR *η*_ρ_^2^=0.00
CpG 2	0.814 (0.079)	13	0.738 (0.086)	8	F(1,19)=4.30, *P*=0.052^†^, *η*_ρ_^2^=0.19	F(3,17)=4.56, *P*=0.049*, *η*_ρ_^2^=0.21	Age *η*_ρ_^2^=0.00, WtHR *η*_ρ_^2^=0.04
CpG 4/5/6	0.142 (0.074)	13	0.106 (0.043)	9	F(1,20)=1.69, *P*=0.208, *η*_ρ_^2^=0.09	F(3,18)=2.51, *P*=0.131, *η*_ρ_^2^=0.12	Age *η*_ρ_^2^=0.00, WtHR *η*_ρ_^2^=0.20*
CpG 8	0.231 (0.096)	14	0.210 (0.091)	10	F(1,22)=0.28, *P*=0.599, *η*_ρ_^2^=0.01	F(3,20)=0.33, *P*=0.573, *η*_ρ_^2^=0.02	Age *η*_ρ_^2^=0.02, WtHR *η*_ρ_^2^=0.02
CpG 9	0.089 (0.050)	13	0.075 (0.030)	10	F(1,21)=0.63, *P*=0.437, *η*_ρ_^2^=0.03	F(3,19)=0.57, *P*=0.458, *η*_ρ_^2^=0.03	Age *η*_ρ_^2^=0.01, WtHR *η*_ρ_^2^=0.00
CpG 12	0.087 (0.060)	13	0.073 (0.031)	8	F(1,19)=0.39, *P*=0.537, *η*_ρ_^2^=0.02	F(3,17)=0.49, *P*=0.495, *η*_ρ_^2^=0.03	Age *η*_ρ_^2^=0.17, WtHR *η*_ρ_^2^=0.05
Mean	0.367 (0.048)	15	0.322 (0.046)	11	F(1,24) =5.68, *P*=0.025*, *η*_ρ_^2^=0.19	F(3,22)=6.16, *P*=0.021*, *η*_ρ_^2^=0.22	Age *η*_ρ_^2^=0.00, WtHR *η*_ρ_^2^=0.09
							
*CRP*							
CpG 1	0.875 (0.048)	12	0.885 (0.031)	10	F(1,20)=0.32, *P*=0.579, *η*_ρ_^2^=0.02	F(3,18)=0.18, *P*=0.675, *η*_ρ_^2^=0.01	Age *η*_ρ_^2^=0.12, WtHR *η*_ρ_^2^=0.00
CpG 2	0.733 (0.064)	12	0.740 (0.058)	10	F(1,20)=0.06, *P*=0.803, *η*_ρ_^2^=0.00	F(3,18)=0.01, *P*=0.937, *η*_ρ_^2^=0.00	Age *η*_ρ_^2^=0.33, WtHR *η*_ρ_^2^=0.22
CpG 4	0.726 (0.074)	12	0.715 (0.051)	10	F(1,20)=0.15, *P*=0.701, *η*_ρ_^2^=0.01	F(3,18)=0.39, *P*=0.539, *η*_ρ_^2^=0.02	Age *η*_ρ_^2^=0.14, WtHR *η*_ρ_^2^=0.07
Mean	0.717 (0.220)	13	0.709 (0.237)	11	F(1,22)=0.01, *P*=0.934, *η*_ρ_^2^=0.00	F(3,20)=0.01, *P*=0.908, *η*_ρ_^2^=0.00	Age *η*_ρ_^2^=0.01, WtHR *η*_ρ_^2^=0.02
							
*LINE-1*							
CpG 1	0.693 (0.020)	15	0.692 (0.017)	11	F(1,24)=0.00, *P*=0.958, *η*_ρ_^2^=0.00	F(3,22)=0.00, *P*=0.974, *η*_ρ_^2^=0.00	Age *η*_ρ_^2^=0.06, WtHR *η*_ρ_^2^=0.07
CpG 2	0.721 (0.014)	15	0.724 (0.009)	11	F(1,24)=0.32, *P*=0.577, *η*_ρ_^2^=0.01	F(3,22)=0.25, *P*=0.626, *η*_ρ_^2^=0.01	Age *η*_ρ_^2^=0.00, WtHR *η*_ρ_^2^=0.04
CpG 3	0.607 (0.012)	15	0.605 (0.015)	11	F(1,24)=0.20, *P*=0.662, *η*_ρ_^2^=0.01	F(3,22)=0.24, *P*=0.632, *η*_ρ_^2^=0.01	Age *η*_ρ_^2^=0.00, WtHR *η*_ρ_^2^=0.04
Mean	0.674 (0.014)	15	0.674 (0.012)	11	F(1,24)=0.00, *P*=0.997, *η*_ρ_^2^=0.00	F(1,22)=0.00, *P*=0.982, *η*_ρ_^2^=0.00	Age *η*_ρ_^2^=0.02, WtHR *η*_ρ_^2^=0.07

Abbreviations: ANCOVA, analysis of covariance; CRP, C-reactive protein; IL-6, interleukin-6; TNF, tumour necrosis factor.

*P<0.05; ^†^*P*<0.10 (two-tailed tests). *η*_ρ_^2^=partial eta squared.

**Table 5:** Results of Spearman's rank-order correlation

	*K10*	*PSS*	*SWB*	*PA*	*hsCRP*	*IL6*	*TNF*	*IL6*_*1*_	*IL6*_*2*_	*TNF*	*CRP*	*LINE-1*
*Questionnaires*												
1. K10	—											
2. PSS	0.654**	—										
3. SWB	−0.445*	−0.128	—									
4. PA	−0.097	−0.273	−0.025	—								
												
*Proteins*												
5. hsCRP	0.089	0.337^†^	0.113	0.199	—							
6. IL6	−0.061	0.040	−0.188	0.001	−0.003	—						
7. TNF	−0.034	0.245	−0.028	−0.149	0.059	0.608^**^	—					
												
*DNA methylation*												
8. IL6_1_	−0.031	0.096	0.400^†^	−0.099	−0.119	0.069	0.312	—				
9. IL6_2_	0.006	0.082	0.290	−0.093	0.121	−0.219	−0.107	−0.020	—			
10. TNF	0.242	0.154	0.046	−0.011	0.001	−0.222	0.017	0.129	0.038	—		
11. CRP	−0.237	−0.053	0.364^†^	−0.398^†^	−0.031	−0.214	0.021	0.126	0.323^†^	0.211	—	
12. LINE-1	0.126	0.409*	−0.125	−0.382^†^	0.202	−0.114	−0.057	−0.008	0.074	0.053	0.384^†^	—

Abbreviations: hsCRP, high-sensitivity C-reactive protein; IL, interleukin; K10, psychological distress; PA, positive affect; PSS, Perceived Stress Scale; SWB, subjective well-being; TNF, tumour necrosis factor.

**P<0.01; *P<0.05; ^†^*P*<0.10 (two-tailed tests).
