# Supplementary material for: Preliminary indications of the effect of a brief yoga intervention on markers of inflammation and DNA methylation in chronically stressed women
Source: Transl Psychiatry. 2016 Nov 29;6(11):e965–. doi: 10.1038/tp.2016.234 (PMC5290356; doi:10.1038/tp.2016.234)
Supplement: Supplementary Information [file tp2016234x1.docx]

**SUPPLEMENTARY MATERIAL**

**Additional Statistical Analysis.**

**Supplementary Table 1. Analysis of Longitudinal IL-6_1_ Methylation**

|  |  | All samples included | | | | Only samples assayed in triplicate within 10% of the individual’s mean | | | |
| --- | --- | --- | --- | --- | --- | --- | --- | --- | --- |
|  |  | CpG 1 | CpG 2 | CpG 4/5/6 | Mean | CpG 1 | CpG 2 | CpG 4/5/6 | Mean |
|  | N | 9 | 9 | 9 | 9 | 6 | 3 | 6 | 9 |
| Pre-Tx | Mean | 0.894 | 0.914 | 0.923 | 0.909 | 0.889 | 0.914 | 0.92 | 0.909 |
|  | SE | 0.007 | 0.011 | 0.005 | 0.003 | 0.006 | 0.012 | 0.01 | 0.003 |
| Post-Tx | Mean | 0.879 | 0.887 | 0.911 | 0.892 | 0.884 | 0.927 | 0.93 | 0.701 |
|  | SE | 0.009 | 0.025 | 0.015 | 0.015 | 0.011 | 0.012 | 0.01 | 0.133 |
| Effect | Cohen's d | 0.63 | 0.46 | 0.38 | 0.53 | 0.21 | 0.60 | 0.36 | 0.74 |
| t-test | P | .236 | .281 | .457 | .252 |  |  |  |  |

**p < .01; *p < .05; †p <.10 (two-tailed tests); Pre-Tx = the averaged pre-intervention methylation (Wave 2 and Wave 3); Post-Tx = post-intervention methylation (Wave 4). Significance tests were not applied for reduced sample sizes due to low Ns.

**Supplementary Table 2. Analysis of Longitudinal IL-6_2_ Methylation**

|  |  | All samples included | | | | | Only samples assayed in triplicate within 10% of the individual’s mean | | | | |
| --- | --- | --- | --- | --- | --- | --- | --- | --- | --- | --- | --- |
|  |  | CpG 1 | CpG 2 | CpG 4/5/6 | CpG 7/8 | Mean^a^ | CpG 1 | CpG 2 | CpG 4/5/6 | CpG 7/8 | Mean |
|  | N | 10 | 10 | 10 | 10 | 10 | 9 | 9 | 9 | 9 | 10 |
| Pre-Tx | Mean | 0.032 | 0.006 | 0.028 | 0.053 | 0.034 | 0.031 | 0.006 | 0.028 | 0.068 | 0.034 |
|  | SE | 0.002 | 0.002 | 0.003 | 0.004 | 0.003 | 0.002 | 0.002 | 0.003 | 0.007 | 0.003 |
| Post-Tx | Mean | 0.034 | 0.002 | 0.034 | 0.064 | 0.034 | 0.032 | 0.002 | 0.037 | 0.067 | 0.031 |
|  | SE | 0.004 | 0.002 | 0.007 | 0.010 | 0.004 | 0.004 | 0.002 | 0.007 | 0.010 | 0.005 |
| Effect | Cohen's d | 0.22 | 0.63 | 0.38 | 0.47 | <0.00 | 0.14 | 0.68 | 0.53 | 0.06 | 0.20 |
| t-test | P | .520 | .138 | .283 | .260 | .988 |  |  |  |  |  |

**p < .01; *p < .05; †p <.10 (two-tailed tests); Pre-Tx = the averaged pre-intervention methylation (Wave 2 and Wave 3); Post-Tx = post-intervention methylation (Wave 4). Significance tests were not applied for reduced sample sizes due to low Ns.

**Supplementary Table 3. Analysis of Longitudinal TNF Methylation**

|  |  | All samples included | | | | | | | Only samples assayed in triplicate within 10% of the individual’s mean | | | | | | |
| --- | --- | --- | --- | --- | --- | --- | --- | --- | --- | --- | --- | --- | --- | --- | --- |
|  |  | CpG 1 | CpG 2 | CpG 4/5/6 | CpG 8 | CpG 9 | CpG 12 | Mean | CpG 1 | CpG 2 | CpG 4/5/6 | CpG 8 | CpG 9 | CpG 12 | Mean |
|  | N | 9 | 9 | 9 | 9 | 9 | 9 | 9 | 1 | 2 | 4 | 5 | 5 | 4 | 8 |
| Pre-Tx | Mean | 0.849 | 0.788 | 0.118 | 0.209 | 0.075 | 0.072 | 0.357 | 0.910 | 0.838 | 0.130 | 0.240 | 0.075 | 0.069 | 0.358 |
|  | SE | 0.016 | 0.035 | 0.018 | 0.026 | 0.011 | 0.015 | 0.011 | . | 0.043 | 0.038 | 0.039 | 0.008 | 0.023 | 0.012 |
| Post-Tx | Mean | 0.733 | 0.730 | 0.077 | 0.159 | 0.070 | 0.048 | 0.302 | 0.700 | 0.810 | 0.078 | 0.158 | 0.090 | 0.040 | 0.200 |
|  | SE | 0.046 | 0.034 | 0.008 | 0.015 | 0.015 | 0.012 | 0.011 |  | 0.050 | 0.017 | 0.027 | 0.025 | 0.011 | 0.035 |
| Effect | Cohen's d | 1.11 | 0.55 | 1.00 | 0.80 | 0.12 | 0.59 | 1.68 | N/A | 0.42 | 0.90 | 1.09 | 0.37 | 0.79 | 2.06 |
| t-test | P | .042* | .241 | .019* | .203 | .806 | .130 | .002** |  |  |  |  |  |  |  |

**p < .01; *p < .05; †p <.10 (two-tailed tests); Pre-Tx = the averaged pre-intervention methylation (Wave 2 and Wave 3); Post-Tx = post-intervention methylation (Wave 4). Significance tests were not applied for reduced sample sizes due to low Ns.

**Supplementary Table 4. Analysis of Longitudinal CRP Methylation**

|  |  | All samples included | | | | Only samples assayed in triplicate within  10% of the individual’s mean | | | |
| --- | --- | --- | --- | --- | --- | --- | --- | --- | --- |
|  |  | CpG 1 | CpG 2 | CpG 4 | Mean | CpG 1 | CpG 2 | CpG 4 | Mean |
|  | N | 9 | 9 | 9 | 9 | 4 | 4 | 4 | 7 |
| Pre-Tx | Mean | 0.870 | 0.720 | 0.692 | 0.761 | 0.893 | 0.723 | 0.703 | 0.606 |
|  | SE | 0.020 | 0.023 | 0.016 | 0.012 | 0.028 | 0.011 | 0.023 | 0.079 |
| Post-Tx | Mean | 0.899 | 0.688 | 0.697 | 0.757 | 0.880 | 0.723 | 0.645 | 0.763 |
|  | SE | 0.021 | 0.043 | 0.031 | 0.022 | 0.041 | 0.065 | 0.056 | 0.029 |
| Effect | Cohen's d | 0.47 | 0.31 | 0.06 | 0.07 | 0.2 | 1.2 | 0.67 | 1.00 |
| t-test | P | .392 | .466 | .916 | .892 |  |  |  |  |

**p < .01; *p < .05; †p <.10 (two-tailed tests); Pre-Tx = the averaged pre-intervention methylation (Wave 2 and Wave 3); Post-Tx = post-intervention methylation (Wave 4). Significance tests were not applied for reduced sample sizes due to low Ns.

**Supplementary Table 5. Analysis of Longitudinal Global Methylation (LINE 1).**

|  |  | CpG 1 | CpG 2 | CpG 3 | Mean |
| --- | --- | --- | --- | --- | --- |
|  | N | 10 | 10 | 10 | 10 |
| Pre-Tx | Mean | .684 | .716 | .602 | .667 |
|  | SE | .004 | .002 | .003 | .003 |
| Post-Tx | Mean | .681 | .711 | .596 | .662 |
|  | SE | .009 | .006 | .005 | .006 |
| Effect | Cohen's d | 0.12 | 0.36 | 0.49 | 0.32 |
| t-test | *p* | .801 | .440 | .348 | .523 |

**p < .01; *p < .05; †p <.10 (two-tailed tests); Pre-Tx = the averaged pre-intervention methylation (Wave 2 and Wave 3); Post-Tx = post-intervention methylation (Wave 4). Note all samples were successfully assayed in triplicate within 10% of the mean.

**Supplementary Table 6. Genetic regions and primer sequences analysed for DNA methylation analysis.**

| Genetic locus | Primer type | Primer sequence | Target size (bp) | No. of detectable CpG sites  (over no. of units) |
| --- | --- | --- | --- | --- |
| *IL6_1_* | Forward  Reverse | aggaagagagGAGATATTATTTTGAGGGAAGAGGG  cagtaatacgactcactatagggagaaggctACCTACATAAACCCCAAATCTCCTA | 309 | 6 (3) |
| *IL6_2_* | Forward  Reverse | aggaagagagTAGGATTGGAGATGTTTGAGGTTTA  cagtaatacgactcactatagggagaaggctAACAACACAACTAAAAACCTACCTCT | 234 | 7 (4) |
| *TNF-α* | Forward  Reverse | aggaagagagTTTGGTTTTTAAAAGAAATGGAGGT  cagtaatacgactcactatagggagaaggctACTTCTCTCCCTCTTAACTAATCCTC | 410 | 8 (6) |
| *CRP* | Forward  Reverse | aggaagagagTTTTAATATTGTTTGTTGGGGTAGG  cagtaatacgactcactatagggagaaggctCATCTCCAAAAACTATCAAATTTCC | 314 | 3 (3) |
| LINE-1 | Forward  Reverse | aggaagagagTTTATATTTTGGTATGATTTTGTAG  cagtaatacgactcactatagggagaaggctTCACCACCAAACCTACCCTAAA | 103 | 3 (3) |

**Supplementary Table 7. PCR reaction protocol for all assays**

| **Reagent** | **Volume for 1 well (µl)** |
| --- | --- |
| 2x FastStart PCR Master Mix | 7.5 |
| Forward primer (10µM) | 0.6 |
| Reverse primer (10µM) | 0.6 |
| Nuclease-free water | 5.3 |
| DNA (25ng/µl) | 1 |
| **Total** | **15.0** |

**Supplementary Table 8. PCR conditions protocol for *IL-6, TNF*, and *CRP***

| **Stage** | **Cycles** | **Temperature** | **Time** |
| --- | --- | --- | --- |
| 1 | 1 | 95°C | 10 minutes |
| 2 | 5 | 95°C  54-60°C  72°C | 10 seconds  30 seconds  2 minutes |
| 3 | 40 | 95°C  58-62°C  72°C | 10 seconds  30 seconds  1.5 minutes |
| 4 | 1 | 72°C  4°C | 7 minutes  ∞ |

**Supplementary Table 9. PCR conditions protocol for *LINE-1***

| **Step** | **Temperature (°C )** | **Time** | **Cycle** | **Concept** |
| --- | --- | --- | --- | --- |
| 1 | 95 | 10 mins | 1 | Denaturation |
| 2 a | 95 | 10 seconds | 5 | Denaturation |
| b | 58 | 30 seconds |  | Annealing |
| c | 72 | 2 mins |  | Extension |
| 3 a | 95 | 10 seconds | 30 | Denaturation |
| b | 61 | 30 seconds |  | Annealing |
| c | 72 | 90 seconds |  | Extension |
| 4 | 72 | 7 minutes | 1 | Extension |
| 5 | 4 | ∝ | ∝ | Finish |
